# Increased difficulty accessing food and income change during the COVID-19 pandemic among youth living in the eThekwini district, South Africa

**DOI:** 10.1017/S1368980024001174

**Published:** 2024-05-23

**Authors:** Julie Jesson, Bongiwe Zulu, Kalysha Closson, C Andrew Basham, Mags Beksinska, Erica Dong, Campion Zharima, Rishav Singh, Tatiana Pakhomova, Janan Dietrich, Angela Kaida

**Affiliations:** 1 Center for Epidemiology and Research in POPulation Health (CERPOP), Inserm, Université de Toulouse, Université Paul Sabatier, Toulouse, France; 2 MRU (MatCH Research Unit), Department of Obstetrics and Gynaecology, Faculty of Health Sciences, University of the Witwatersrand, Durban, South Africa; 3 Center of Gender Equity and Health, University of California San Diego, La Jolla, CA, USA; 4 Faculty of Health Sciences, Simon Fraser University, Burnaby, BC, Canada; 5 Division of Pharmacoepidemiology and Pharmacoeconomics, Department of Medicine, Brigham and Women’s Hospital, Boston, MA, USA; 6 Department of Medicine, Harvard Medical School, Boston, MA, USA; 7 Perinatal HIV Research Unit (PHRU), Faculty of Health Sciences, University of the Witwatersrand, Johannesburg, South Africa; 8 African Social Sciences Unit of Research and Evaluation (ASSURE), Faculty of Health Sciences, University of the Witwatersrand, Johannesburg, South Africa; 9 African Social Sciences Unit of Research and Evaluation (ASSURE), Wits Health Consortium, Johannesburg, South Africa; 10 Health Systems Research Unit, South African Medical Research Council, Bellville, South Africa; 11 Women’s Health Research Institute, Vancouver, BC, Canada

**Keywords:** COVID-19, Young adult, Food security, Income, Developing countries

## Abstract

**Objective:**

To estimate the effect of income change on difficulty accessing food since the COVID-19 pandemic for South African youth and evaluate whether this effect was modified by receiving social grants.

**Design:**

A cross-sectional, online survey was conducted between December 2021 and May 2022. Primary outcome was increased difficulty accessing food since the COVID-19 pandemic. Income change was categorised as ‘Decreased a lot’, ‘Decreased slightly’ and ‘Unchanged or increased’. Multivariable logistic regressions were used, with an interaction term between social grant receipt and income change.

**Setting:**

eThekwini district, South Africa.

**Participants:**

Youth aged 16–24 years.

**Results:**

Among 1,620 participants, median age was 22 years (IQR 19–24); 861 (53 %) were women; 476 (29 %) reported increased difficulty accessing food; 297 (18 %) reported that income decreased a lot, of whom 149 (50 %) did not receive social grants. Experiencing a large income decrease was highly associated with increased difficulty accessing food during the COVID-19 pandemic (adjusted OR [aOR] 3·63, 95 % CI 2·70, 4·88). The aOR for the effect of a large income decrease on difficulty accessing food, compared to no income change, were 1·49 (95 % CI 0·98, 2·28) among participants receiving social grants, and 6·63 (95 % CI 4·39, 9·99) among participants not receiving social grants.

**Conclusions:**

While social grant support made a great difference in lowering the effect of income decrease on difficulty accessing food, it was insufficient to fully protect youth from those difficulties. In post-pandemic recovery efforts, there is a critical need to support youth through economic empowerment programming and food schemes.

The COVID-19 pandemic has had a negative, sustained impact on the global food system, disrupting food production, supply and access^(1)^. Prior to, and since, the COVID-19 pandemic, geopolitical instability and climate change continue to disrupt access to adequate, sufficient and nutritious food^(2)^. It is estimated that the COVID-19 pandemic led to an additional 137 million people suffering from food insecurity in 2020^(3)^. People experienced food shortages, resulting in uncertainty about their ability to obtain food. They were forced to compromise on the quality and/or quantity of the food they consume, and many went days without eating. The increase in food insecurity since the COVID-19 pandemic is worrying as food access and healthy nutritional intake play a major role on general health and well-being, both in the short and long term^(4)^, and are key factors of adolescent physical, cognitive and social development^(5)^. Extensive literature has documented the role of food insecurity on affecting mental health outcomes^(6)^ and on increasing gender-based violence^(7)^.

Individuals who face the greatest difficulties accessing food are more frequently those with lower educational level and poor socio-economic backgrounds, whatever the country income group^(8)^. In South Africa, the majority of the population lives under the poverty line, with a 32·7 % unemployment rate in the country in 2022^(9)^. South Africa has also one of the highest HIV prevalence rates in the world, and people living with HIV commonly experience high rates of food insecurity and malnutrition^(10)^, that reflect their socio-economic vulnerability. In this context, with multiple existing threats that could lead to food insecurity, the COVID-19 pandemic and the public health mitigation measures implemented to contain the pandemic constitutes an additional burden to access to food.

While sub-Saharan Africa may have reported lower COVID-19 mortality rates than higher income settings^(11,12)^, this region experienced tremendous indirect social, economic and health consequences of the COVID-19 public health mitigation measures, with major access disruptions to healthcare, education and workplaces^(12–14)^. The pandemic also contributed to increases in gender-based violence^(15)^ as well as mental health issues, globally and in sub-Saharan Africa^(16)^. Among sub-Saharan Africa countries, South Africa has reported the highest number of COVID-19 cases (an estimated 4 million by July 2023) and deaths (an estimated 102 000 by July 2023)^(17)^. A national lockdown was declared in South Africa between 27 March and 1 May 2020, which was considered the most restrictive lockdown in sub-Saharan Africa, and also one of the most restrictive worldwide^(18)^. This lockdown included a stay-at-home order, gathering restrictions, alcohol and tobacco sale bans, as well as travel restrictions, unless for essential reasons, with movements between provinces and districts prohibited. South Africans were confined to their homes, and all forms of public transport were prohibited. Exceptions were possible only when seeking essential services, emergency or chronic medical attention, for funeral services, or for collecting a social grant or pension, with capacity limits. All non-essential businesses were ordered to close, and retail stores selling essential goods were prohibited from selling any other goods. The National School Nutrition Programme (NSNP), which provides 9 million meals per d for learners, was interrupted^(19,20)^. These circumstances have been most impactful on the financial security and food access of South Africa’s poorest communities, leading to riots, protests and confrontations with police^(18,21)^. After this first lockdown period, South Africa started to ease the restrictions as it moved through five lockdown levels^(22)^, but, for many, the disruptions on education, employment, access to care and food remain^(23)^. Few studies have described the difficulties to accessing food during and after the restrictions or the role of the related mitigation measures that led to work disruptions and economic loss.

Adolescents and young adults have been among those most affected by social and economic disruptions related to the COVID-19 pandemic, with school closures and lack of job opportunities during a crucial time of their career development. However, little is known about the impacts of the COVID-19 pandemic on this population in South Africa^(24,25)^. Despite food access and healthy nutritional intake being crucial components of their physical, cognitive and social development^(26)^, specific experiences of youth related to food access during the COVID-19 pandemic are lacking. Food insecurity and hunger were two of the main concerns reported in a qualitative survey among South African youth in April 2020 regarding their COVID-19 experiences^(20)^, showing that COVID-19 was already perceived as a major threat for food access.

Difficulties in accessing food during the pandemic in South Africa have been reported by the National Income Dynamics Study – Coronavirus Rapid Mobile Survey (NIDS-CRAM), a nationally representative panel survey conducted between April 2020 and May 2021^(27)^. In April 2020, 47 % of households reported ‘having run out of money to buy food’, which was twice as high as in 2017, with one-third (35 %) continuing to experience this problem in March 2021. In May 2021, household hunger (17 %) and child hunger (14 %) remained high, with significant, sustained increases among households who experienced job loss due to the COVID-19 lockdown^(28)^. Thus, over a year after the beginning of the COVID-19 pandemic and the implementation of control measures, many South African households remained economically vulnerable and severely food-insecure^(29)^.

To tackle the socio-economic effects of the COVID-19 pandemic through mitigation measures, the South African government extended its social grants programme to include a range of revised and new social support grants. A special COVID-19 Social Relief of Distress (SRD) grant was introduced in April 2020, giving the equivalent of R350 (about USD21 in 2020) per month for working age adults >18 years who were unemployed and not receiving any other grant, until April 2021^(30)^. There were also top-ups for households receiving the Child Support Grant (R480 per month + top-up of R240), received by over 7 million caregivers, and top-ups of R250 for the Old Age Pension Grant for people over 60 years of age (R1990 per month), the Disability Grant (maximum R2080 per month) and the Foster Care Grant (R1070 per month). Several studies have analysed the role of these social grants in reducing household and child hunger, with varying results^(28,31)^. However, few studies have focused on the social and economic impacts of the COVID-19 pandemic among adolescents and young adults^(24,25)^.

We described the increased difficulty accessing food since the COVID-19 pandemic for South African youth and investigated how changes in income since the COVID-19 pandemic affected access to food and if receiving social grant moderates the relationship between income change and increased difficulty accessing food. We hypothesised that the COVID-19 pandemic and associated public health mitigation measures led to further difficulties in accessing food among youth, especially through economic disruptions. A secondary hypothesis was that financial support from the government may have contributed to moderate the detrimental effects of these economic disruptions on the difficulty accessing food.

## Methods

### Study design and population

AYAZAZI RIGHTS (Rapid Investigation of Gendered Health outcomes in the Time of SARS-CoV-2) was a cross-sectional survey examining South African youth experiences during the COVID-19 public health response. This online survey took place in the eThekwini district, in KwaZulu-Natal, a province that have been especially affected by the COVID-19 pandemic, with the higher proportion of youth in the country, and the highest average household size (4·4 people in 2022)^(32)^. Among the 1 125 765 households censused in 2016, 3001 were headed by youth under 18 years old^(33)^. The survey was conducted from 21 December 2021 to 31 May 2022, a time period when South Africa experienced a fourth COVID-19 wave, due to the newly discovered Omicron variant, with a level 1 alert (including mandatory mask indoor and capacity limits for gatherings). The National State of Disaster was lifted on 15 April 2022. Inclusion criteria included: youth aged 16–24 years; residing in the eThekwini district, Durban, South Africa; and ability to read in English and/or isiZulu and have access to the Internet via a mobile phone, tablet or computer. For this analysis, we restricted the analytic sample to participants with non-missing data regarding access to food, income change and covariates.

### Recruitment

A multi-pronged recruitment strategy was used to enrol participants. First, we approached participants from previous youth studies led by the University of the Witwatersrand MatCH Research Unit (Wits MRU, based in Durban), who agreed to be recontacted for other research projects via text messages. Second, we engaged with the Wits MRU Adolescent Community Advisory Board (ACAB), the adult Wits MRU’s Community Advisory Board (CAB) and other community-based and youth-led organisations serving youth across eThekwini district to share information about the study via e-mails and through their social networks. Third, we distributed recruitment flyers in areas highly frequented by youth such as commercial retail settings, transit areas and healthcare facilities. The survey link was also displayed on the Wits MRU website as a pop-up message and was posted on social media platforms of youth-led organisations, including a not-for-profit company located in Durban that specialises in media communication access for vulnerable communities in South Africa. Participants also had the opportunity to share the link and the QR code of the survey with their eligible peers and family members. To further promote the survey, a prize draw to win cash prizes of R100 (CAD$ 8·50) was offered for participants providing their mobile phone number at the end of the survey (and unlinked with survey responses). Overall, a total of 269 cash prizes were given out, corresponding to a 10 % chance of winning over the enrolment period. Participants could enter and complete the mobile health survey via a free web link provided via the #datafree Moya Messenger App.

### Data collection and analytic sample

**Fig. 1:**
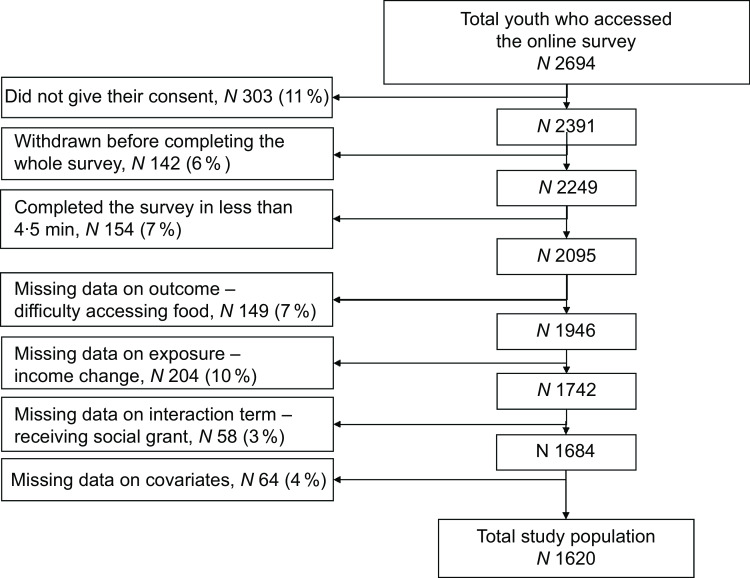
Selection of the study population – AYAZAZI RIGHTS 2022

The questionnaire was auto-administered and structured with close-ended questions across four survey sections: demographics; COVID-19 experiences (including COVID-19 vaccine uptake and perceptions, changes in income, work disruptions, access to food and social grants); sexual and reproductive health, care, and behaviours; and mental health, care, and substance use. The questionnaire was created in English and translated in isiZulu, with back-translation into English to ensure accuracy. The questionnaire was programmed into REDCap (Research Electronic Data Capture)^(34)^ and piloted with youth and topical experts with revisions integrated prior to the study launch. Overall, the median time of completion was 15 min (interquartile range [IQR] = 8–26). Participants who completed the whole questionnaire in less than 4·5 min were subsequently excluded from the analyses as this was considered as a too short time to fully read, understand and complete it. Overall, 2694 youth accessed the online survey. Among them, 303 (11 %) did not consent to participate and 142 (6 %) did not complete the whole survey. Among the 2249 participants that completed the whole survey, we excluded 154 (7 %) who completed it in less than 4·5 min. Subsequently, we excluded 149 (7 %) participants with missing data on food access difficulty, then 204 (10 %) with missing data on income change, 58 (3 %) with missing data on receiving social grant and finally 64 (4 %) participants with missing data on covariates (including gender, ethnicity, household structure and HIV status), leading to a final study population of 1620 (Fig. 1).

### Measures

The primary outcome was difficulty accessing food during the COVID-19 pandemic, measured using the following question: ‘Since the COVID-19 pandemic, did your access to sufficient, quality food change?’ with four response options: ‘It was more difficult to access food’, ‘It has not changed’, ‘It was easier to access food’ and ‘Prefer not to answer’. Responses were dichotomised to enable comparison between participants who reported increased difficulty accessing food *v.* participants who did not, as less than 1 % of the whole sample reporting that their access to food was easier. Those who responded ‘Prefer not to answer’ were not included. Additionally, we descriptively explored other aspects of difficulty accessing food, by collecting further information about work disruptions among those employed and about suggested factors limiting food access using multiple-choice questions.

The primary exposure of interest hypothesised to affect food access was COVID-19-related economic disruptions. Economic disruptions were assessed by measuring income change since the COVID-19 pandemic, categorised as ‘Decreased a lot’, ‘Decreased slightly’, ‘Unchanged’, ‘Increased slightly’ and ‘Increased a lot’. Due to small cell count for the last two categories (<5 %), there were combined with the ‘Unchanged’ group, into one reference category, for further analyses.

The interaction term of interest was receiving any social grant from the government since the COVID-19 pandemic, whether personally or for their household. Additionally, information about the type of social grant received was asked, that is, whether the grant was for child support, old age pension, disability, foster child or another purpose. The ‘other’ category was subsequently recoded as all participants specified that ‘other grant’ referred to the COVID-19 SRD grant. Changes in social grant support since the COVID-19 pandemic (i.e. increased, stayed the same, decreased or do not know) were also reported.

Potential confounders were identified based on previously published literature^(35)^ as sociodemographic factors known to be associated with food insecurity and difficulty to access food. Gender was described in three categories: boy/man, girl/woman and gender non-conforming (i.e. self-identifying as non-binary or not identifying with any gender). Ethnicity was categorised using the official population groups from South Africa Census^(33)^: Black African, Coloured, White, Indian and Asian (in the South African context, Coloured refers to ‘any person of “mixed blood” as well as descendants from Black–White, Black–Asian, White–Asian, and Black–Colored unions’)^(36)^. Classifications of Black African and Colored are not racial but rather social constructs of South African historical apartheid origin^(37)^. In addition, we accounted for income level (under or above R800 [about USD48 in 2020] per month), being enrolled at school and/or employed (if no to both, participants were defined as Neither in Education, Employment or Training [NEET]^(38)^), the structure of the household including the number of children (age 0–17 years), adults (18–59) and seniors (from age 60 years) living in the household (reported as numerical variables by participants and described as binary variables for each category), having children (none, one, two or more) and self-reported HIV status (positive, negative, unsure of HIV status or prefer not to say).

### Statistical analyses

Characteristics of the study population were described overall and stratified by the outcome variable (increased difficulty accessing food). Characteristics were also compared between the population included and the population excluded due to missing data. Additionally, social grant support (received or not, type and change) was described overall and stratified by income change. These descriptions were further stratified by gender: men, women and gender non-conforming (see online supplementary material, Supplementary Tables). Multivariable logistic regression models estimated the association between income decrease (whether a lot or slightly) and increased difficulty accessing food during the COVID-19 pandemic, adjusted by the covariates listed above. To assess potential modification of the effect of an income decrease on the difficulty accessing food by receipt of social grant support, an interaction term was included for income change by social grant receipt. Subgroup analyses by gender were also conducted. Data analyses were performed using SAS® OnDemand for Academics, © 2022 SAS Institute Inc.

## Results

### Participant characteristics

Among the 1620 participants, median age was 22 years (IQR 19–24); 53 % were women, 5 % gender non-conforming and 76 % were Black-African. Median size of the household was seven people (IQR 5–9), 19 % of participants were living with two or more seniors in their household and 41 % were living with three or more children. Only 1 % of participants were living by themselves. Overall, 476 (29 %) participants reported that it was more difficult to access food since the COVID-19 pandemic. Since the COVID-19 pandemic, 18 % reported their income had decreased a lot and 14 % reported their income had decreased slightly; 68 % had no income change. Overall, 35 % reported having received, personally and/or by someone else in their household, social grants from the government. Among participants who reported increased difficulty accessing food, 34 % reported that their income decreased a lot, and 53 % of them were receiving social grant support (Table 1a). When comparing the included population with the excluded population due to missing data (*n* 475), no differences were found in the distribution for the main exposure, interaction terms and covariates. However, a higher rate of participants in the excluded population reported that it was more difficult to access food since the COVID-19 pandemic (38 %).

**Table 1a:** Characteristics of the study population according to the increased difficulty to access food since the COVID-19 pandemic. AYAZAZI RIGHTS online survey, eThekwini district, South Africa, 2022

Variables			Increased difficulty accessing food			
	Overall *n* 1620		Yes, *n* 476		No, *n* 1144	
*Main exposure variables*						
Income change since the COVID-19 pandemic						
Decreased a lot	297	18·3 %	160	33·6 %	137	12·0 %
Decreased slightly	219	13·5 %	58	12·2 %	161	14·1 %
Unchanged or increased	1104	68·1 %	258	54·2 %	846	74·0 %
Received social grant support						
Yes	565	34·9 %	250	52·5 %	315	27·5 %
No	1055	65·1 %	226	47·5 %	829	72·5 %
*Covariates*						
Gender						
Man	681	42·0 %	178	37·4 %	503	44·0 %
Woman	861	53·1 %	276	58·0 %	585	51·1 %
Non-conforming	78	4·8 %	22	4·6 %	56	4·9 %
Age groups (years)						
16–18	405	25·0 %	76	16·0 %	329	28·8 %
19–24	1215	75·0 %	400	84·0 %	815	71·2 %
Ethnicity						
Black African	1233	76·1 %	372	78·2 %	861	75·3 %
Coloured, Indian or Asian	318	19·6 %	91	19·1 %	227	19·8 %
White	69	4·3 %	13	2·7 %	56	4·9 %
Having children						
None	1046	64·6 %	239	50·2 %	807	70·5 %
One	463	28·6 %	179	37·6 %	284	24·8 %
Two or more	111	6·9 %	58	12·2 %	53	4·6 %
Current occupation, part-time or full time						
At school and employed	86	5·3 %	31	6·5 %	55	4·8 %
At school only	744	45·9 %	158	33·2 %	586	51·2 %
Employed only	323	19·9 %	116	24·4 %	207	18·1 %
Neither at school or employed	467	28·8 %	171	35·9 %	296	25·9 %
Income level						
No income–R800	695	42·9 %	239	50·2 %	456	39·9 %
R800 or more	925	57·1 %	237	49·8 %	688	60·1 %
Number of adults living in household						
None to 3	657	40·6 %	203	42·6 %	454	39·7 %
4 or more	963	59·4 %	273	57·4 %	690	60·3 %
Number of seniors living in household						
None or 1	1310	80·9 %	343	72·1 %	967	84·5 %
2 or more	310	19·1 %	133	27·9 %	177	15·5 %
Number or children aged 0–17 years living in household						
None, 1 or 2	954	58·9 %	223	46·8 %	731	63·9 %
3 or more	666	41·1 %	253	53·2 %	413	36·1 %
HIV status						
HIV-positive	86	5·3 %	44	9·2 %	42	3·7 %
HIV-negative	1077	66·5 %	267	56·1 %	810	70·8 %
Unsure of HIV status or prefer not to say	457	28·2 %	165	34·7 %	292	25·5 %

Receiving social grant support differed by income change: among participants reporting that their income decreased a lot, 50 % received social grant support, while among participants reporting that their income decreased slightly, 39 % of them received social grant support. Among participants who received social grant support, 76 % of them were receiving Child Support Grant, and 48 % were receiving Old Age Pension Grant. Only 3 % of participants reported having received the COVID-19 SRD grant. Furthermore, among participants who received social grant support, 49 % of them saw their support increased since the COVID-19 pandemic, while 31 % did not know if this support has changed (Table 1b).

**Table 1b:** Social grant support received, overall and according to income change. AYAZAZI RIGHTS online survey, eThekwini district, South Africa, 2022

Social grant support			According to income change					
	Total (*n* 1620)		Decreased a lot (*n* 297)		Decreased slightly (*n* 219)		Unchanged or increased (*n* 1104)	
Have you or your household received any social grants from the government since the COVID-19 pandemic?								
Yes	565	34·9 %	149	50·2 %	85	38·8 %	331	30·0 %
No	1055	65·1 %	148	49·8 %	134	61·2 %	773	70·0 %
* If yes, what social grants did you receive?*								
Child Support Grant	429	75·9 %	109	73·2 %	55	64·7 %	265	80·1 %
Old Age Pension Grant	269	47·6 %	39	26·2 %	40	47·1 %	190	57·4 %
Disability Grant	53	9·4 %	15	10·1 %	8	9·4 %	30	9·1 %
Foster Child Grant	39	6·9 %	11	7·4 %	4	4·7 %	24	7·3 %
COVID-19 Social Relief of Distress grant	14	2·5 %	4	2·7 %	1	1·2 %	9	2·7 %
* If yes, did your social grants support from the government change since the COVID-19 pandemic?*								
Support increased	276	48·8 %	69	46·3 %	29	34·1 %	178	53·8 %
Support stayed the same	73	12·9 %	18	12·1 %	18	21·2 %	37	11·2 %
Support decreased	36	6·4 %	20	13·4 %	11	12·9 %	5	1·5 %
I don’t know	174	30·8 %	42	28·2 %	27	31·8 %	105	31·7 %
Missing data	6	1·1 %	0	0·0 %	0	0·0 %	6	1·8 %

### Effects of income change on difficulty accessing food

The crude estimated OR for the effect of a large income decrease (‘Decreased a lot’), compared with no change in income, on difficulty accessing food was 3·83 (95 % CI 2·93, 5·00), and the covariate-adjusted OR (aOR) was 3·63 (95 % CI 2·70, 4·88). The strength of this association was slightly higher for men (aOR 5·05, 95 % CI 3·10, 8·22) than women (aOR 3·29, 95 % CI 2·19, 4·93). The crude OR for a slight income decrease (‘Decreased slightly’), compared with no change in income, on difficulty accessing food was 1·18 (95 % CI 0·85, 1·64) overall, and the aOR was 1·37 (95 % CI 0·95, 1·96). Similarly, the strength of this association was higher for men (aOR 2·18, 95 % CI 1·26, 3·76) than women (aOR 1·14, 95 % CI 0·67, 1·92) (Table 2).

**Table 2:** Estimated adjusted OR between income change and increased difficulty accessing food, overall and by gender. Logistic regression models. RIGHTS online survey, eThekwini district, South Africa, 2022

Analyses	Total sample (*N* 1620)		Men (*n* 681)		Women (*n* 861)		Gender non-conforming (*n* 78)	
	(a)OR	95 % CI	(a)OR	95 % CI	(a)OR	95 % CI	aOR	95 % CI
Crude estimated OR								
Income decreased a lot *v*. unchanged or increased	3·83	2·93, 5·00	5·71	3·69, 8·84	2·84	1·99, 4·05	4·89	1·46, 16·36
Income decreased slightly *v*. unchanged or increased	1·18	0·85, 1·64	2·03	1·24, 3·34	0·79	0·49, 1·27	1·04	0·24, 4·44
Covariate-adjusted estimated OR								
Income decreased a lot *v*. unchanged or increased	3·63	2·70, 4·88	5·05	3·10, 8·22	3·29	2·19, 4·93	5·98	1·38, 25·80
Income decreased slightly *v*. unchanged or increased	1·37	0·95, 1·96	2·18	1·26, 3·76	1·14	0·67, 1·92	1·15	0·25, 5·35

aOR, adjusted OR.Covariate-adjusted models for the total sample, men and women are adjusted for age group, gender (except for models stratified by gender) ethnicity, having children, current occupation, income level, household structure (number of adults, children and seniors living in the same household) and HIV status. Covariate-adjusted model for gender non-conforming participants adjusted only on age group, current occupation, income level and household structure to ensure model fit.

**Fig. 2:**
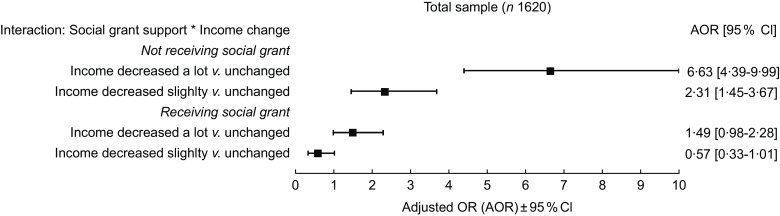
Estimated adjusted OR between income change and increased difficulty accessing food, according to receiving social grant support. Multivariable logistic regression model. RIGHTS online survey, eThekwini district, South Africa, 2022

When looking at the effect of income change on difficulty accessing food according to receiving social grants, the aOR for the effect of a large income decrease, compared with no change in income, on difficulty accessing food was 1·49 (95 % CI 0·98, 2·28) among participants receiving social grants, and 6·63 (95 % CI 4·39, 9·99) among participants not receiving social grants. The aOR for the effect of a slight income decrease was 0·57 (95 % CI 0·33, 1·01) among participants receiving social grants, and 2·31 (95 % CI 1·45, 3·67) among participants not receiving social grant (Fig. 2). Similar modification effects of receiving social grants were found by gender (Appendix 2).

### Limiting factors to access food and work disruptions

**Fig. 3:**
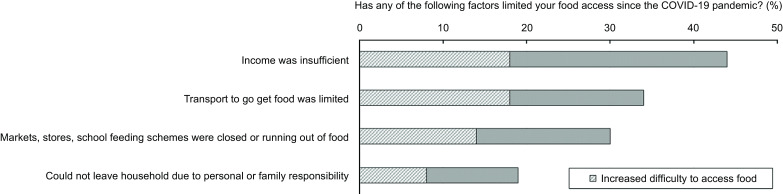
Factors limiting food access since the COVID-19 pandemic (*n* 1620). Overall and according to increased difficulty accessing food. AYAZAZI RIGHTS online survey, eThekwini district, South Africa, 2022

**Fig. 4:**
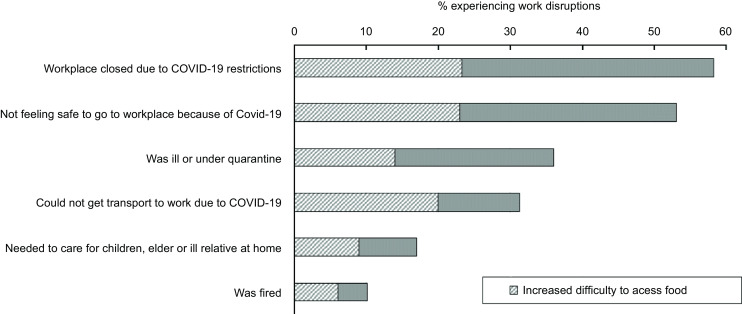
Experiences of work disruptions for those employed (*n* 409). Overall and according to increased difficulty accessing food. AYAZAZI RIGHTS online survey, eThekwini district, South Africa, 2022

When asking for factors that may have limited their food access during the COVID-19 pandemic, 44 % of participants declared it was because of insufficient income, 34 % because transport to go get food was limited, 30 % because food markets, stores or school feeding schemes were either closed or running out of food and 19 % because they could not leave their household due to personal or family responsibilities. Participants who faced any of these limiting factors were more likely to report increased difficulties accessing food during the COVID-19 pandemic (between 42 % and 52 %) (Fig. 3). Among those who were employed (*n* 409), multiple experiences of work disruptions and employee challenges since the onset of the COVID-19 pandemic were reported, such as workplace closures (59 %). Additionally, 53 % reported not feeling safe to go to their workplace, 36 % were ill or under quarantine, 32 % could not get transport to work, 16 % needed to care for children or relatives and 10 % were fired. All of these experiences were related to increased difficulty to access food, especially being fired (63 %) and not getting transport to work (64 %) (Fig. 4).

## Discussion

In this cross-sectional study, we found that one-third of South African youth surveyed, of all genders, experienced increased difficulty to access sufficient, quality food since the onset of the COVID-19 pandemic in March 2020. These results were partially explained by the indirect effects of the COVID-19 pandemic on the economy, with those youth experiencing income loss having higher odds of experiencing greater difficulty accessing food during the COVID-19 pandemic. This was especially the case for youth who did not receive any social grants, who had over six times higher odds of having difficulty accessing food when experiencing a large income decrease. While receiving social grants partly mitigated the association between income loss and increased difficulty accessing food, there were still 49 % higher odds of difficulty in accessing food due to a large income decrease.

Previous studies have described the limited effects of social grant support in mitigating the economic impacts of the COVID-19 pandemic on outcomes such as mental health in Canada and France^(39)^ or severe food insecurity in Nigeria^(40)^. In South Africa, within the NIDS-CRAM study, having received a social grant (either the Child Support Grant, the SRD grant or the Old Age Pension Grant)^(41)^ was associated with a decrease in household and child hunger by 17–24 %^(29,41)^. A substantial decline in the prevalence of hunger was recorded between May and August 2020, a time when a greater relaxation of lockdown restrictions was observed, but also a period when the government social grants programme increased as part of the pandemic response^(42)^. Studies point out that this government social grants programme was inadequate, with top-ups and special grants implemented during COVID-19 lockdowns being insufficient to reduce household and especially child hunger. In particular, it was reported that the social grant programme did not adequately target women, partly because women who already received child grants could not apply for the SRD grant^(30)^. In our survey, only one-third of participants reported receiving social grants, and only 50 % of those who experienced large income loss received social grant support. Most of the social grant support came from child support or old age pension grants. These youth were likely living in households that receive social grants but did not receive these grants themselves.

Very few participants declared receiving the COVID-19 SRD grant, which may be partly explained by the lack of a specific response option for this grant. Rather, survey respondents specified receipt of the SRD as an ‘Other’ grant response. A report from the Department of Social Development in South Africa, assessing the implementation and utilisation of the SRD grant, found that this grant was mostly used to purchase food^(30)^. However, this report also highlights the difficulties to access this grant for people in need, as there were a lot of grant’s criteria to fulfil, considered as exclusionary and disadvantageous. The application for this grant was to be completed online, excluding people without access to online services and with a low level of digital literacy. Others have described that the communication on how to access and apply to this grant was considered insufficient and not enough targeted for people in need^(30)^. In this context, many youth who could have been eligible to this grant did not apply due to lack of means, limited Internet access and/or knowledge. Therefore, youth may not have received the adequate financial support to mitigate the socio-economic impacts of the pandemic, such as the impact on food access. The lockdown measures limited youth from moving about and earning additional sources of income for themselves and their families (e.g. informal markets, day labour, etc) that could have helped mitigate the insufficient social grant support and food access difficulties. Youth living in households with younger children had also to deal with the interruption of the NSNP during the lockdown, with the additional challenge to feed those children who did not get access to meals at school during this period^(43)^.

Our study also provides a description of the multiple work disruptions encountered by South African youth who were employed at the onset of the COVID-19 pandemic, such as workplace closures, transport disturbances and not feeling safe to go to work because of COVID-19. Among this minority of youth who were employed, the pandemic had devastating effects on their ability to maintain their employment and sufficiently provide for themselves and their families, with work disruptions having highly affected their ability to access food. As the baseline level of unemployment was already excessively high among youth (58 % among 15–24 years in 2019)^(44)^, the COVID-19 pandemic has emphasised and worsen the existing challenges faced by South African youth in getting non-precarious jobs^(45)^. In our study, youth who experience work disruptions were particularly affected by increased difficulty accessing food, which is in line with the challenges reported by a similar population of youth in a 2020 qualitative study^(20)^.

Difficulties in accessing food during the COVID-19 pandemic increased equally for men, women and gender non-conforming participants in our survey, but this does not exclude that the baseline level of food insecurity prior to the COVID-19 pandemic response in March 2020 may have been different by gender. The NIDS-CRAM survey described a higher level of food insecurity among South African women compared to men during the COVID-19 pandemic^(42)^. This may be due to women having been especially affected by the socio-economic impacts of the COVID-19 pandemic^(46)^, representing two of the three million of job losses recorded in April 2020 by NIDS-CRAM^(47)^. Studies conducted prior to the COVID-19 pandemic showed higher level of food insecurity for South African women compared to men, especially for women-headed households^(48)^. This can be explained by the socio-economic challenges faced by women in South Africa, where gender norms, roles and behaviours remain strongly marked^(49)^, and women are disproportionately represented in informal employment^(49)^. Few studies have focused on food insecurity among South African youth while exploring gender differences. A previous survey conducted between 2014 and 2016 among youth living in Soweto and Durban found similar food insecurity rates for young men and woman^(35)^.

This study has limitations. The cross-sectional study design limits the interpretation and the assessment of the causality between large income decrease and increased difficulty accessing food. Data collection occurred over an extended period of time during the COVID-19 pandemic (March 2020–May 2022), which went across several different alert levels. Survey distribution took place during a level 1 alert period, which may have led to participants omitting some of their experiences over those last 2 years, or only shared the most recent or difficult ones. This change in alert levels may have influenced participants’ responses in reporting difficulty accessing food. Responses may have, therefore, been subject to recall bias. Because participants who were excluded from the analysis due to missing data reported a higher rate of increased difficulty accessing food than the study population (38 % *v*. 29 %), it is important to consider the possibility of selection bias and underestimation of the importance of food insecurity in this context. Furthermore, it is also possible that responses may have been subject to social desirability bias. However, such bias may have been minimised by the use of an anonymous online survey format. Comparisons with other studies are limited due to the use of non-standardised questions, especially for the study outcome of food insecurity. More robust measures, that better take into account the complexity of food security, consisting in scoring multiple questions, exist (such as the nine-item Household Food Insecurity Access Scale, or the three-item Household Hunger Scale) but were not used for our study due to the necessity to keep the survey questionnaire short, as well as the fact that these measures are especially designed to assess current food insecurity profiles (i.e. in the last month), and not necessarily for an extended period of time like in our study (i.e. March 2020–May 2022). We were, however, able to adjust our analyses on a range of relevant sociodemographic characteristics which are known confounders of the association between economic hardship and food insecurity. Information regarding the modalities of food access for youth (i.e. responsible for purchasing their own food or not, or purchasing for their household) could have been of additional value for this study. Despite using a convenience sample, we developed a multi-pronged recruitment approach, including working in collaboration with community partners, to capture a large and diverse sample of South African youth living in the eThekwini district in Durban, a district that has been especially affected by the COVID-19 pandemic. A key strength is that this survey represents to our knowledge one of the few large quantitative surveys focusing on youth living in sub-Saharan Africa during the COVID-19 pandemic^(50)^.

In conclusion, South African youth have been highly affected by the COVID-19 pandemic, facing socio-economic challenges which were associated with increased difficulty to access food. Few were able to access to social grant support. Our results highlight that regardless of whether youth received social grants, we observed that youth whose income decreased a lot during the pandemic had significantly reduced ability to access food during the pandemic. The critically high effect among those not receiving social grants signals a need to support youth in accessing financial supports during ongoing and future public health crises. While social grant support made a great difference in lowering the effect of income decrease on difficulty accessing food, it was insufficient to fully protect youth from those difficulties. Our data highlight the need to develop adapted financial and non-financial support for this underserved group, especially in the event of further public health crises. In light of the continued recovery from the pandemic and for future pandemic responses, there is a critical need to support youth through economic empowerment programming and food security policy and planning.

## Supporting information

Jesson et al. supplementary material 1 Jesson et al. supplementary material

## Supplementary material

For supplementary material accompanying this paper visit https://doi.org/10.1017/S1368980024001174.
