# Supplementary material for: Increased difficulty accessing food and income change during the COVID-19 pandemic among youth living in the eThekwini district, South Africa
Source: Public Health Nutr. 2024 May 23;27(1):e142. doi: 10.1017/S1368980024001174 (PMC11617416; doi:10.1017/S1368980024001174)
Supplement: Jesson et al. supplementary material 1 — Jesson et al. supplementary material [file S1368980024001174sup001.docx]

**Appendix 1a: Characteristics of the study population according to the increased difficulty to access food since the COVID-19 pandemic, stratified by gender. AYAZAZI RIGHTS online survey, eThekwini District, South Africa, 2022**

| **Variables** | **Men N=681** | | **Increased difficulty**  **accessing food** | | | |  | **Women N=861** | | **Increased difficulty**  **accessing food** | | | |  | **Gender non-conforming N=78** | | **Increased difficulty accessing food *** | | | |
| --- | --- | --- | --- | --- | --- | --- | --- | --- | --- | --- | --- | --- | --- | --- | --- | --- | --- | --- | --- | --- |
|  |  |  | **Yes,**  **N=178** | | **No,**  **N=503** | |  |  |  | **Yes,**  **N= 276** | | **No,**  **N=585** | |  |  |  | **Yes,**  **N=22** | | **No,**  **N= 56** | |
| ***Main exposure variables*** | | | |  |  |  |  |  |  |  |  |  |  |  |  |  |  |  |  |  |
| **Income change since the COVID-19 pandemic** | | | | |  |  |  |  |  |  |  |  |  |  |  |  |  |  |  |  |
| Decreased a lot | 115 | 16.9% | 64 | 36.0% | 51 | 10.1% |  | 166 | 19.3% | 87 | 31.5% | 79 | 13.5% |  | 16 | 20.5% | 9 | 40.9% | 7 | 12.5% |
| Decreased slightly | 94 | 13.8% | 29 | 16.3% | 65 | 12.9% |  | 111 | 12.9% | 26 | 9.4% | 85 | 14.5% |  | 14 | 17.9% | - | - | - | - |
| Unchanged or increased | 472 | 69.3% | 85 | 47.8% | 387 | 76.9% |  | 584 | 67.8% | 163 | 59.1% | 421 | 72.0% |  | 48 | 61.5% | 10 | 45.5% | 38 | 67.9% |
| **Received social grant support** | | |  |  |  |  |  |  |  |  |  |  |  |  |  |  |  |  |  |  |
| Yes | 212 | 31.1% | 89 | 50.0% | 123 | 24.5% |  | 337 | 39.1% | 153 | 55.4% | 184 | 31.5% |  | 22 | 28.2% | 11 | 50.0% | 11 | 19.6% |
| No | 475 | 69.8% | 92 | 51.7% | 383 | 76.1% |  | 524 | 60.9% | 123 | 44.6% | 401 | 68.5% |  | 56 | 71.8% | 11 | 50.0% | 45 | 80.4% |
| ***Covariates*** |  |  |  |  |  |  |  |  |  |  |  |  |  |  |  |  |  |  |  |  |
| **Age groups (years)** |  |  |  |  |  |  |  |  |  |  |  |  |  |  |  |  |  |  |  |  |
| 16-18 | 195 | 28.6% | 34 | 19.1% | 161 | 32.0% |  | 206 | 23.9% | 41 | 14.9% | 165 | 28.2% |  | 4 | 5.1% | - | - | - | - |
| 19-24 | 487 | 71.5% | 144 | 80.9% | 343 | 68.2% |  | 655 | 76.1% | 235 | 85.1% | 420 | 71.8% |  | 74 | 94.9% | 21 | 95.5% | 53 | 94.6% |
| **Ethnicity** |  |  |  |  |  |  |  |  |  |  |  |  |  |  |  |  |  |  |  |  |
| Black African | 534 | 78.4% | 138 | 77.5% | 396 | 78.7% |  | 633 | 73.5% | 216 | 78.3% | 417 | 71.3% |  | 66 | 84.6% | 18 | 81.8% | 48 | 85.7% |
| Coloured, Indian or Asian | 123 | 18.1% | 34 | 19.1% | 89 | 17.7% |  | 184 | 21.4% | 54 | 19.6% | 130 | 22.2% |  | 11 | 14.1% | - | - | - | - |
| White | 24 | 3.5% | 6 | 3.4% | 18 | 3.6% |  | 44 | 5.1% | 6 | 2.2% | 38 | 6.5% |  | 1 | 1.3% | - | - | - | - |
| **Having children** |  |  |  |  |  |  |  |  |  |  |  |  |  |  |  |  |  |  |  |  |
| None | 476 | 69.9% | 106 | 59.6% | 370 | 73.6% |  | 497 | 57.7% | 113 | 40.9% | 384 | 65.6% |  | 73 | 93.6% | 20 | 90.9% | 53 | 94.6% |
| One | 167 | 24.5% | 58 | 32.6% | 109 | 21.7% |  | 293 | 34.0% | 121 | 43.8% | 172 | 29.4% |  | 3 | 3.8% | - | - | - | - |
| Two or more | 38 | 5.6% | 14 | 7.9% | 24 | 4.8% |  | 71 | 8.2% | 42 | 15.2% | 29 | 5.0% |  | 2 | 2.6% | - | - | - | - |
| **Current occupation, part-time or full time** | | | | |  |  |  |  |  |  |  |  |  |  |  |  |  |  |  |  |
| At school and employed | 32 | 4.7% | 10 | 5.6% | 22 | 4.4% |  | 53 | 6.2% | 21 | 7.6% | 32 | 5.5% |  | 1 | 1.3% | - | - | - | - |
| At school only | 330 | 48.5% | 66 | 37.1% | 264 | 52.5% |  | 374 | 43.4% | 82 | 29.7% | 292 | 49.9% |  | 40 | 51.3% | 10 | 45.5% | 30 | 53.6% |
| Employed only | 148 | 21.7% | 49 | 27.5% | 99 | 19.7% |  | 161 | 18.7% | 63 | 22.8% | 98 | 16.8% |  | 14 | 17.9% | - | - | - | - |
| Neither at school or employed | 171 | 25.1% | 53 | 29.8% | 118 | 23.5% |  | 273 | 31.7% | 110 | 39.9% | 163 | 27.9% |  | 23 | 29.5% | 8 | 36.4% | 15 | 26.8% |
| **Income level** |  |  |  |  |  |  |  |  |  |  |  |  |  |  |  |  |  |  |  |  |
| No income -R800 | 309 | 45.4% | 92 | 51.7% | 217 | 43.1% |  | 362 | 42.0% | 138 | 50.0% | 224 | 38.3% |  | 24 | 30.8% | 9 | 40.9% | 15 | 26.8% |
| R800 or more | 372 | 54.6% | 86 | 48.3% | 286 | 56.9% |  | 499 | 58.0% | 138 | 50.0% | 361 | 61.7% |  | 54 | 69.2% | 13 | 59.1% | 41 | 73.2% |
| **Number of adults living in household** | | |  |  |  |  |  |  |  |  |  |  |  |  |  |  |  |  |  |  |
| None to 3 | 286 | 42.0% | 85 | 47.8% | 201 | 40.0% |  | 343 | 39.8% | 110 | 39.9% | 233 | 39.8% |  | 28 | 35.9% | 8 | 36.4% | 20 | 35.7% |
| 4 or more | 395 | 58.0% | 93 | 52.2% | 302 | 60.0% |  | 518 | 60.2% | 166 | 60.1% | 352 | 60.2% |  | 50 | 64.1% | 14 | 63.6% | 36 | 64.3% |
| **Number of seniors living in household** | | |  |  |  |  |  |  |  |  |  |  |  |  |  |  |  |  |  |  |
| None or 1 | 544 | 79.9% | 119 | 66.9% | 425 | 84.5% |  | 699 | 81.2% | 205 | 74.3% | 494 | 84.4% |  | 67 | 85.9% | 19 | 86.4% | 48 | 85.7% |
| 2 or more | 137 | 20.1% | 59 | 33.1% | 78 | 15.5% |  | 162 | 18.8% | 71 | 25.7% | 91 | 15.6% |  | 11 | 14.1% | - | - | - | - |
| **Number or children 0-17 years living in household** | | | | |  |  |  |  |  |  |  |  |  |  |  |  |  |  |  |  |
| None, 1 or 2 | 413 | 60.6% | 82 | 46.1% | 331 | 65.8% |  | 496 | 57.6% | 128 | 46.4% | 368 | 62.9% |  | 45 | 57.7% | 13 | 59.1% | 32 | 57.1% |
| 3 or more | 268 | 39.4% | 96 | 53.9% | 172 | 34.2% |  | 365 | 42.4% | 148 | 53.6% | 217 | 37.1% |  | 33 | 42.3% | 9 | 40.9% | 24 | 42.9% |
| **HIV status** |  |  |  |  |  |  |  |  |  |  |  |  |  |  |  |  |  |  |  |  |
| HIV-positive | 18 | 2.6% | 9 | 5.1% | 9 | 1.8% |  | 66 | 7.7% | 33 | 12.0% | 33 | 5.6% |  | 2 | 2.6% | - | - | - | - |
| HIV-negative | 421 | 61.8% | 85 | 47.8% | 336 | 66.8% |  | 608 | 70.6% | 171 | 62.0% | 437 | 74.7% |  | 48 | 61.5% | 11 | 50.0% | 37 | 66.1% |
| Unsure or prefer not to say | 242 | 35.5% | 84 | 47.2% | 158 | 31.4% |  | 187 | 21.7% | 72 | 26.1% | 115 | 19.7% |  | 28 | 35.9% | 9 | 40.9% | 19 | 33.9% |

*When the number of gender non-conforming participants was <15 by category, we choose to not display the detailed description according to increased difficulty accessing food to protect participants’ identities.

**Appendix 1b: Social grant support received, overall and according to income change, stratified by gender. AYAZAZI RIGHTS online survey. eThekwini District. South Africa. 2022**

| **Social grant support** | **Men (n=681)** | | **According to income change** | | | | | |  | **Women (n=861)** | | **According to income change** | | | | | |  | **Gender non-conforming (n=78)*** | |
| --- | --- | --- | --- | --- | --- | --- | --- | --- | --- | --- | --- | --- | --- | --- | --- | --- | --- | --- | --- | --- |
|  |  |  | **Decreased a lot (n=115)** | | **Decreased slightly (n=94)** | | **Did not change (n=472)** | |  |  |  | **Decreased a lot (n=166)** | | **Decreased slightly (n=111)** | | **Did not change (n=584)** | |  |  |  |
| **Have you or your household received any social grants from the government since the COVID-19 pandemic?** | | | | | | | | | | | | | | | | | | | |  |
| Yes | 206 | 30.2% | 55 | 47.8% | 30 | 31.9% | 121 | 25.6% |  | 337 | 39.1% | 84 | 50.6% | 50 | 45.0% | 203 | 34.8% |  | 22 | 28.2% |
| No | 475 | 69.8% | 60 | 52.2% | 64 | 68.1% | 351 | 74.4% |  | 524 | 60.9% | 82 | 49.4% | 61 | 55.0% | 381 | 65.2% |  | 56 | 71.8% |
|  |  |  |  |  |  |  |  |  |  |  |  |  |  |  |  |  |  |  |  |  |
| ***If yes, what social grants did you receive?*** | | | | | | |  |  |  |  |  |  |  |  |  |  |  |  |  |  |
| Child support | 150 | 72.8% | 38 | 69.1% | 18 | 60.0% | 94 | 77.7% |  | 264 | 78.3% | 63 | 75.0% | 34 | 68.0% | 167 | 82.3% |  | 15 | 68.2% |
| Old age pension | 110 | 53.4% | 15 | 27.3% | 13 | 43.3% | 82 | 67.8% |  | 151 | 44.8% | 22 | 26.2% | 25 | 50.0% | 104 | 51.2% |  | 8 | 36.4% |
| Disability grant | 28 | 13.6% | 10 | 18.2% | 5 | 16.7% | 13 | 10.7% |  | 24 | 7.1% | 5 | 6.0% | 2 | 4.0% | 17 | 8.4% |  | 1 | 4.5% |
| Foster child grant | 19 | 9.2% | 6 | 10.9% | 2 | 6.7% | 11 | 9.1% |  | 19 | 5.6% | 4 | 4.8% | 2 | 4.0% | 13 | 6.4% |  | 1 | 4.5% |
| COVID-19 grant* | 6 | 2.9% | 2 | 3.6% | 1 | 3.3% | 3 | 2.5% |  | 8 | 2.4% | 2 | 2.4% | 0 | 0.0% | 6 | 3.0% |  | 15 | 68.2% |
|  |  |  |  |  |  |  |  |  |  |  |  |  |  |  |  |  |  |  |  |  |
| ***If yes, did your social grants support from the government changed since the COVID-19 pandemic?*** | | | | | | | | | | | | | | | | | |  |  |  |
| Support increased | 68 | 33.0% | 16 | 29.1% | 5 | 16.7% | 47 | 38.8% |  | 202 | 59.9% | 50 | 59.5% | 22 | 44.0% | 130 | 64.0% |  | 6 | 27.3% |
| Support stayed the same | 32 | 15.5% | 11 | 20.0% | 7 | 23.3% | 14 | 11.6% |  | 40 | 11.9% | 7 | 8.3% | 11 | 22.0% | 22 | 10.8% |  | 1 | 4.5% |
| Support decreased | 15 | 7.3% | 9 | 16.4% | 3 | 10.0% | 3 | 2.5% |  | 20 | 5.9% | 10 | 11.9% | 8 | 16.0% | 2 | 1.0% |  | 1 | 4.5% |
| I don't know | 88 | 42.7% | 19 | 34.5% | 15 | 50.0% | 54 | 44.6% |  | 72 | 21.4% | 17 | 20.2% | 9 | 18.0% | 46 | 22.7% |  | 14 | 63.6% |
| Missing data | 3 | 1.5% | 0 | 0.0% | 0 | 0.0% | 3 | 2.5% |  | 3 | 0.9% | 0 | 0.0% | 0 | 0.0% | 3 | 1.5% |  | 0 | 0.0% |

* Due to very small sample size, results for gender non-conforming participants were not displayed according to income change.

**
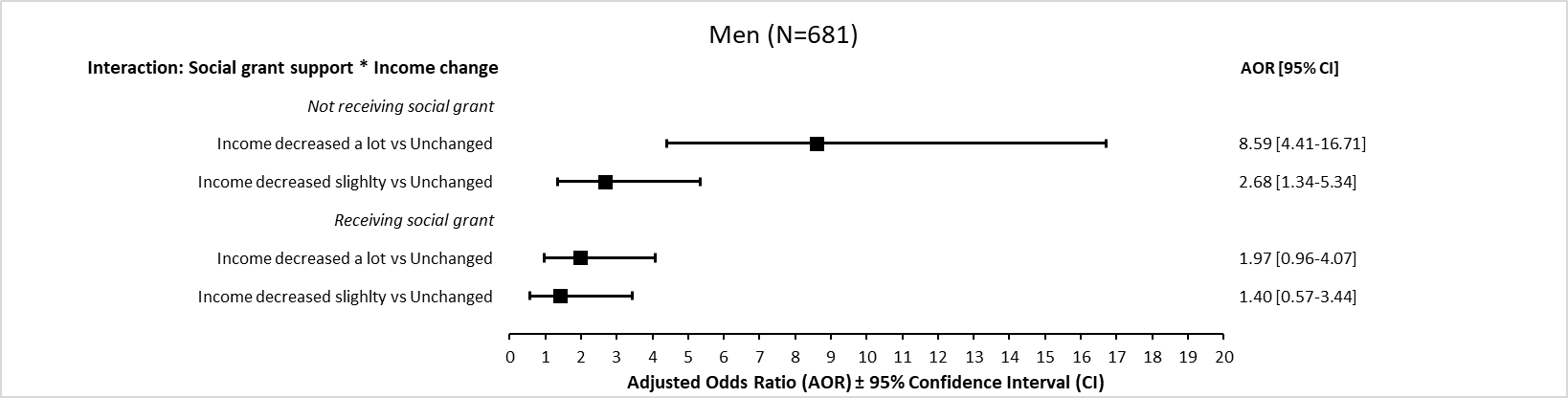
**

**
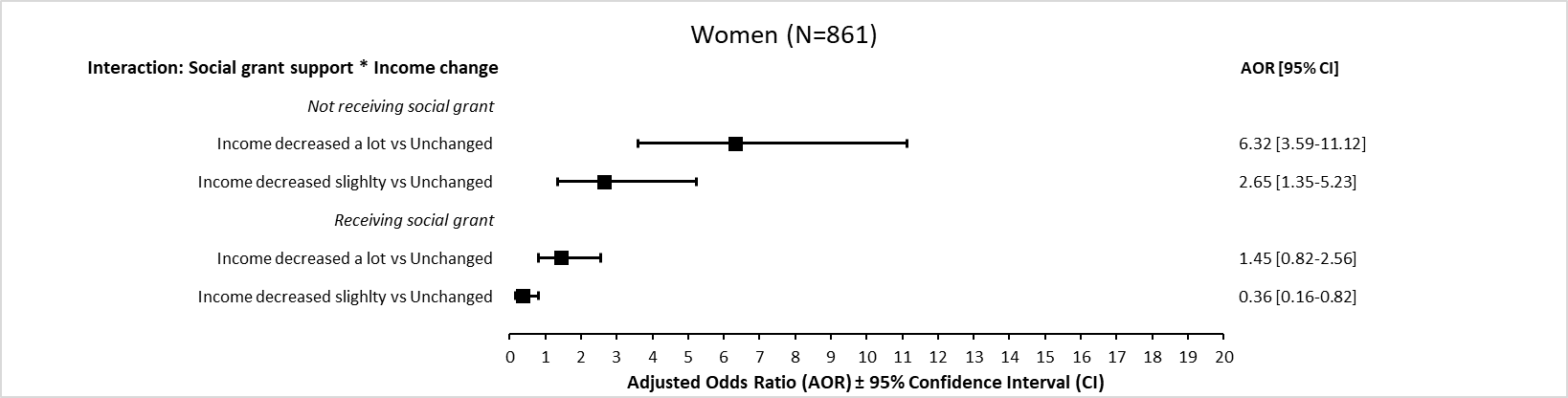
**

**
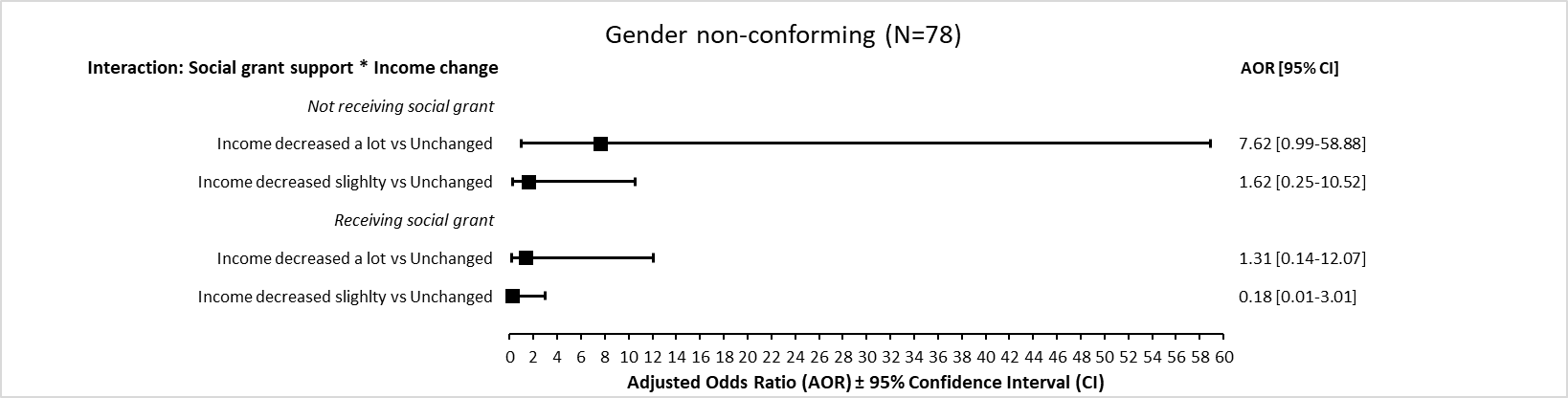
**

**Appendix 2: Estimated adjusted Odds ratio between income change and increased difficulty accessing food, according to receiving social grant support, by gender. Multivariable logistic regression model. RIGHTS online survey. eThekwini District. South Africa. 2022. Abbreviations:** AOR= Adjusted Odds Ratio, CI= 95% Confidence Interval **Notes:** Models for men and women are adjusted for age group, ethnicity, having children, current occupation, income level, household structure (number of adults, children and seniors living in the same household), and HIV status. Model for gender non-conforming participants is adjusted only on age group, current occupation, income level and household structure to ensure model fit.
